# Study on Synergistic Viscosity Reduction Mechanism and Product Characteristics of Co-Aquathermolysis of Corn Stalk and Furfural Extraction Oil

**DOI:** 10.3390/ma19020428

**Published:** 2026-01-22

**Authors:** Qingmei Tian, Zinan Liu, Wenqiang Liu, Yansheng Liu, Xingying Lan, Xiaoling Xu

**Affiliations:** 1 College of Chemical Engineering and Environment, China University of Petroleum (Beijing), Beijing 102249, China; tqm@cupk.edu.cn (Q.T.); wsuper@cup.edu.cn (Y.L.); 2 State Key Laboratory of Heavy Oil Processing, China University of Petroleum (Beijing) at Karamay, Karamay 834000, China; 15811385563@163.com; 3 Refining and Chemical Research Institute, PetroChina Karamay Petrochemical Company Limited, Karamay 834000, China; 18209902736@163.com; 4 State Key Laboratory of Heavy Oil Processing, China University of Petroleum (Beijing), Beijing 102249, China; lanxy@cup.edu.cn

**Keywords:** furfural extraction oil, biomass, aquathermolysis, polycyclic aromatic hydrocarbons

## Abstract

Furfural extraction oil (FEO) is rich in polycyclic aromatic hydrocarbons (PAHs) and is hard to convert under mild conditions. To address this upgrade challenge, this study proposed a co-aquathermolysis process with corn stalk and a Ni/Mo hydrofining catalyst. Key parameters, including reaction temperature, time, catalyst dosage, and corn stalk dosage, were systematically evaluated for their impact on upgrade performance. Under optimized conditions (oil-to-water mass ratio 2:1, 280 °C, 18 h, 8 wt% catalyst, 8 wt% corn stalk), a viscosity reduction rate of 19.96% was achieved, significantly exceeding the 12.69% rate obtained without corn stalk. Meanwhile, the average molecular weight decreased from 430.0 to 353.3 g·mol^−1^ and the aromatic ring index declined from 3.049 to 2.593. The H/C ratio increased to 1.568, and the sulfur content decreased to 0.09210%. ^1^H NMR analysis revealed that corn stalk promotes long-chain scission and inhibits aromatic condensation, leading to a reduced aromatic carbon fraction. A detailed hydrocarbon composition analysis corroborated the conversion of tricyclic and tetracyclic aromatic hydrocarbons to monocyclic and bicyclic aromatic hydrocarbons. These findings offer valuable insights for the modification of FEO via aquathermolysis and establish biomass utilization as a practical strategy for FEO upgrades.

## 1. Introduction

Furfural extraction oil (FEO) is a by-product of the manufacturing process of lubricating oils. It is separated as non-ideal lubricant components that are dissolved in the furfural solvent during the refining of vacuum gas oil or solvent–deasphalted oil [1]. FEO is rich in polycyclic aromatic hydrocarbons (PAHs), including naphthalene-, anthracene-, and phenanthrene-series compounds, together with resins and trace asphaltenes. The total aromatic content typically ranges from 50 to 80%, which is significantly higher than that of straight-run diesel or fluid catalytic cracking slurry, and the aromatics are dominated by species containing two or more fused rings [2]. Because aromatic conjugation confers high chemical stability, the direct conversion of PAHs is challenging. Consequently, FEO is commonly used as a low-value heavy fuel oil. Nevertheless, FEO holds promise as a valuable aromatic feedstock, in alignment with growing emphasis on resource recycling and green chemical engineering. Advances in conversion technologies now enable its selective processing to recover industrial aromatics such as naphthalene and anthracene, which are used in dye and pharmaceutical intermediate synthesis. Alternatively, FEO can be modified into environmentally benign rubber softeners, substituting for conventional, highly toxic aromatic oils, or they can serve as a precursor for needle coke to mitigate shortages of high-quality carbon materials. However, the current utilization of FEO remains limited by the high energy demands of conventional processes, including hydrocracking for the production of light aromatics. Developing more efficient and less intensive conversion technologies is imperative in order to overcome this bottleneck.

Aquathermolysis technology has garnered extensive utilization in the upgrading of heavy oil in recent years [3,4,5,6,7,8]. Under hydrothermal conditions, the cleavage of C–S, C–C, and C–N bonds can occur, thereby converting heavy components into smaller molecules [9]. In this process, water serves as the reaction medium and a potential hydrogen source [10]. Active hydrogen is formed as a consequence of the water–gas shift (WGS) reaction [11] and water dissociation on the catalyst surface [12]. The cleavage of chemical bonds generates a large number of free radicals during the aquathermolysis reaction. The combination of these free radicals with active hydrogen results in the formation of low-carbon chain hydrocarbons, thereby leading to a reduction in heavy oil viscosity. However, the active hydrogen provided by water is limited, which causes some active chain segments to recombine and form macromolecular high-carbon chain hydrocarbons. This is unfavorable for aquathermolysis. Thus, hydrogen donors should be added to the reaction system to facilitate the combination of free radicals with hydrogen and promote the progress of the reaction.

The efficient utilization of biomass energy can reduce fossil fuel consumption, waste generation, and greenhouse gas emissions. As a renewable energy alternative to fossil fuels, it has attracted increasing attention worldwide [13,14,15]. In co-aquathermolysis, small molecules produced from biomass hydrothermal liquefaction can serve as hydrogen donors and facilitate catalytic upgrade reactions [16]. He Bin et al. [17] investigated the co-upgrading of Bohai heavy oil with biomass such as microcrystalline cellulose and waste paper scraps over an Fe_2_O_3_/Al_2_O_3_ catalyst. They found that the biomass was converted into alcohols, aliphatic hydrocarbons, and aromatic hydrocarbons at the early stage. Aliphatic hydrocarbons and alcohols subsequently generated additional reactive hydrogen on the catalyst surface [18]. This active hydrogen, in synergy with Fe_2_O_3_, promoted C–C and C–S bond cleavage and enhanced deep cracking toward lighter components. Wang et al. [19] studied the co-aquathermolysis of sawdust with Liaohe extra-heavy oil and reported that biomass addition continuously supplied reactive hydrogen, resulting in a viscosity reduction exceeding 90%. Similarly, Lin et al. [20] attributed the synergistic effect in the co-pyrolysis of oily sludge and rice husk primarily to active hydrogen supplied by rice husk, which promoted the conversion of heavy fractions, increased the yields of saturates and aromatics, and reduced heavy components, thereby improving oil quality.

Most studies in the past have focused on conventional heavy oils dominated by resin–asphaltene colloidal structures, whereas FEO is distinguished by its exceptionally high PAH content and the refractory nature of multi-ring aromatics. Resins and asphaltenes in heavy oils exhibit high aromaticity, and their fundamental structural units typically comprise aromatic sheets containing at least three fused rings; asphaltenes generally show even higher aromatic condensation per sheet [21]. This structural feature resembles the PAH-rich composition of FEO, suggesting that aquathermolysis may also be applicable to promoting PAH conversion in FEO. However, aquathermolysis studies specifically targeting FEO remain limited, and investigations coupling FEO upgrades with biomass-assisted hydrogen donation are even scarcer.

This study aimed to investigate the feasibility and effectiveness of a hydrogen supply from corn stalk for upgrading FEO under hydrothermal conditions. The effects of reaction temperature, time, catalyst dosage, and corn stalk dosage on the upgrade performance of FEO were examined. Moreover, the physicochemical properties and compositional changes in FEO before and after aquathermolysis were characterized to elucidate the mechanism of biomass-facilitated upgrades.

## 2. Experimental

### 2.1. Materials

The fourth-side-cut furfural extraction oil used in this study was obtained from the Refining and Chemical Research Institute, PetroChina Karamay Petrochemical Co., Ltd. (Karamay, China), and its basic physical properties are listed in Table 1. Corn stalk biomass was dried and milled to 120 mesh. The cellulose, hemicellulose, and lignin contents in corn stalk were determined using the Van Soest method and are reported as mass percentages on a dry basis [22] (Table 2). An industrial Ni/Mo hydrorefining catalyst (NiO and MoO_3_ supported on Al_2_O_3_) was employed, and its characterization data were taken from our published work [23] (Table 3). Toluene, *n*-heptane, dichloromethane, petroleum ether (90–120 °C), and absolute ethanol were purchased from the Tianjin Zhiyuan chemical reagent factory (Tianjin, China). All other chemicals and solvents were of analytical grade.

### 2.2. Aquathermolysis Experiment

All experiments were performed in a high-pressure and high-temperature batch reactor (HT-250FJ, Shanghai Huotong Experimental Instrument Co., Ltd., Shanghai, China). FEO and deionized water were charged at a fixed oil-to-water mass ratio of 2:1, followed by the addition of the catalyst and corn stalk as required. Before heating, the reactor was purged with nitrogen for 15 min to establish oxygen-free conditions. The heater was then switched on, and the temperature was increased. After the target temperature was reached, the stirring speed was set to 400 rpm to promote phase contact, and the reaction time was recorded. During heating and the reaction, the reactor operated under autogenous pressure, which increased with temperature and was continuously monitored by the built-in pressure sensor. As shown in Figure S1, when the temperature reached 280 °C, the pressure increased to 6.41 MPa and remained nearly constant (typically within ±0.1 MPa) during the isothermal period. Because the stabilized pressure at 280 °C is close to the saturated vapor pressure of water at this temperature, the system is described as operating in a near-saturated vapor–liquid (two-phase) regime, with a dense liquid (hot compressed water/oil) phase coexisting with a vapor headspace. After the reaction, heating was stopped and the reactor was naturally cooled to 80 °C, as no forced cooling control was applied. The oil–water mixture was discharged, and the oil phase was collected by centrifugation at 4000 rpm for 10 min. The catalyst was recovered, rinsed with toluene to remove residual oil and deposits, dried, and sealed for storage. All experiments, including blank runs and biomass-assisted runs, followed the same heating and cooling procedure.

### 2.3. Biomass Hydrothermal Liquefaction Experiment

Firstly, 6.4 g of corn stalk and 40 g of deionized water were weighed and added into a high-temperature and high-pressure reactor (HT-100FJ, Huotong, Shanghai, China). The materials were then thoroughly mixed with a glass rod. Subsequently, the reactor was sealed, and the internal air was purged with nitrogen. Subsequently, the reactor was heated to 280 °C and operated at 400 rpm for the requisite reaction period. Subsequent to the conclusion of the reaction, the mixture was extracted and separated using dichloromethane, thereby yielding bio-oil, an aqueous phase, and solid residues.

### 2.4. Evaluation and Characterization of Furfural Extraction Oil

The viscosity of the oil samples was measured at 40 °C using a rotational viscometer (Anton Paar SVM3000, Shanghai, China) according to ASTM D445 [24]. The viscosity reduction rate was calculated according to Equation (1):

(1) $$\eta (\%)=\frac{\mu_{0}-\mu_{1}}{\mu_{0}}\times 100\%$$

where *ŋ*, *μ*_0_, and *μ*_1_ are the viscosity reduction rate, viscosity before the reaction, and viscosity after the reaction, respectively.

Other physicochemical parameters of the oil samples were determined as follows: molecular weight was measured according to SH/T 0398-2007 [25] using a UIC 833 molecular weight analyzer (Joliet, IL, USA). Group composition was analyzed according to SH/T 0753-2009 [26]. Carbon-type composition was calculated from density (SH/T 0604-2000 [27]), the refractive index (ASTM D1747 [28]), and kinematic viscosity (ASTM D445) following ASTM D2140 [29]. Elemental contents of C, H, N, and S were measured using a UNICUBE elemental analyzer with an instrumental deviation of less than 0.1 wt%. Detailed hydrocarbon composition was analyzed by GC-MS using a Shimadzu LHY-13-S-010 instrument (Kyoto, Japan).

H NMR spectra were acquired on a Bruker Avance-500 spectrometer (Beijing, China) using CDCl_3_ as the solvent and tetramethylsilane (TMS) as the internal standard. Proton assignments for different chemical environments are listed in Table 4.

Based on the improved Brown–Ladner methods, structural parameters such as the aromatic degree (*f*_A_), the aromatic condensation (H_AU_/C_A_), and the branching index (BI) of FEO were calculated according to Equations (2)–(4) [30].

(2) $$f_{A}=\frac{C_{T}/H_{T}-\left(H_{\alpha }+H_{\beta }+H_{\gamma }\right)/2H_{T}}{C_{T}/H_{T}}$$

(3) $$\frac{H_{AU}}{C_{A}}=\frac{H_{A}/H_{T}+H_{\alpha }/2H_{T}}{C_{T}/H_{T}-\left(H_{\alpha }+H_{\beta }+H_{\gamma }\right)/2H_{T}}$$

(4) $$BI=\frac{2H_{\gamma }}{3\left(H_{\alpha }+H_{\beta }\right)}$$

where H_T_ is the sum of H_A_, H*_α_*, H*_β_*, and H*_γ_*. C_T_/H_T_ signifies the molar proportion of carbon to hydrogen, derived from the elemental analysis data previously acquired.

### 2.5. Aqueous Phase Product Characterization

The aqueous phase after the reaction was analyzed by GC-MS (890B–7000C, Agilent Technologies, Santa Clara, CA, USA). Briefly, 5 mL of the aqueous phase was mixed with 10 mL of dichloromethane in a stoppered graduated cylinder. After vigorous shaking and phase separation, 1 mL of the dichloromethane phase was transferred to a sample vial for analysis. The instrument was preheated to 40 °C, increased to 100 °C at 6 °C·min^−1^, then increased to 230 °C at 10 °C·min^−1^ and was held for 3 min.

### 2.6. Catalyst Characterization

FT-IR characterization of the catalyst was performed on a Thermo Nicolet iS5 spectrometer (Thermo Fisher Scientific, Waltham, MA, USA). The surface morphology of the catalyst was examined by FIB-SEM (ZEISS Crossbeam 550, Carl Zeiss, Jena, Germany), and elemental composition was analyzed by energy-dispersive X-ray spectroscopy (EDX; XFlash 6160, Bruker, Berlin, Germany) equipped on the same instrument.

## 3. Results and Discussion

### 3.1. The Impact of Reaction Conditions on the Upgrade of Furfural Extraction Oil

**Figure 1:**
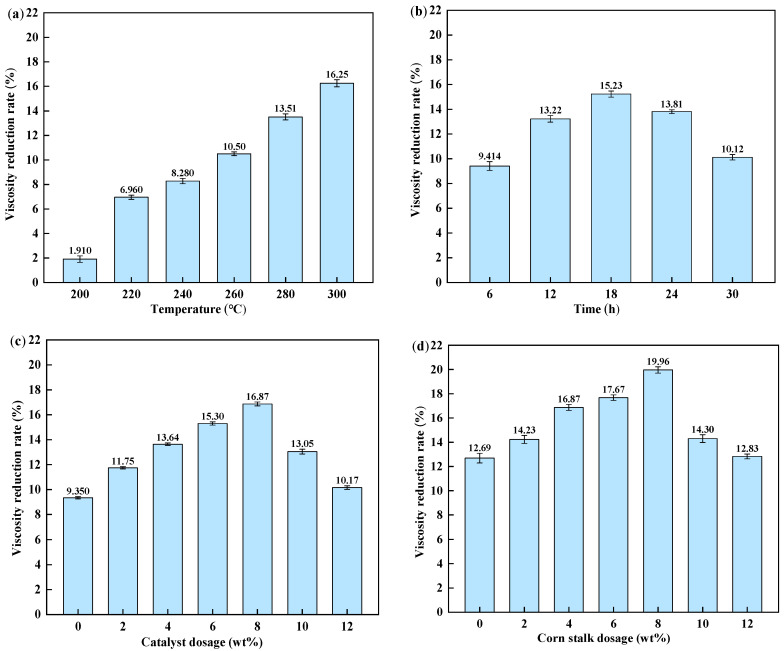
Effect of reaction conditions on viscosity reduction rate of FEO: (**a**) reaction temperature; (**b**) reaction time; (**c**) catalyst dosage; (**d**) corn stalk dosage.

The reaction was conducted with 4 wt% catalyst and 2 wt% corn stalks for 12 h. As illustrated in Figure 1a, the viscosity reduction rate increased with temperature. This trend is attributed to enhanced bond cleavage and improved mass transfer under hydrothermal conditions, together with an increased formation of reactive hydrogen species from water and biomass-derived intermediates [31,32,33]. Further increasing the temperature above 280 °C produced no notable additional viscosity reduction.

In biomass-assisted upgrades of conventional heavy oils, the viscosity reduction rate exceeds 88% within a similar temperature range [34]. The smaller improvement observed here is mainly related to feedstock structure. For typical heavy oils, viscosity is strongly governed by resin/asphaltene colloidal networks that can be disrupted effectively in the presence of hydrogen donors. By contrast, the fourth-side-cut furfural extraction oil contains negligible resins and asphaltenes and is dominated by stable PAHs. Consequently, the scission of fused-ring systems and hydrocracking of condensed aromatics are more difficult under the present conditions, resulting in a moderate viscosity reduction.

Figure 1b shows the viscosity reduction rate at different reaction times (280 °C, 4 wt% catalyst, 2 wt% corn stalk). The viscosity reduction rate increased initially and reached a maximum at 18 h, after which it decreased. This trend suggests that the synergistic effect between the catalyst and corn stalk-derived intermediates is optimized at 18 h. Beyond 18 h, the hydrogen-donating capacity of liquefaction intermediates may become insufficient for the continued deep conversion of refractory components, and partial coverage or deactivation of active sites can occur. As a result, radical recombination and secondary condensation become more competitive, leading to heavier products and a higher viscosity.

To evaluate the influence of catalyst dosage, reactions were conducted at 280 °C for 18 h with a fixed corn stalk dosage of 2 wt%. As shown in Figure 1c, the viscosity reduction rate followed a non-monotonic trend. It initially increased, reaching a maximum at a catalyst dosage of 8 wt%, and then decreased. This optimum is likely due to the catalyst’s effectiveness in removing heteroatoms and enhancing the conversion of heavy components during aquathermolysis.

Figure 1d illustrates the effect of corn stalk dosage at 280 °C for 18 h with 8 wt% catalyst. The viscosity reduction rate increased as the corn stalk dosage increased up to 6.4 g per 80 g of FEO and then decreased at higher dosage. At excessive biomass dosage, a fraction of corn stalk may not be fully liquefied and can remain as a suspended solid, which increases the viscosity. At moderate dosage, biomass liquefaction produces oxygenates and light gases that enhance reactive hydrogen formation and hydrogen transfer reactions, thereby promoting bond cleavage, dealkylation and hydrogenation, and the suppression of secondary condensation. Therefore, an intermediate corn stalk dosage provides the most effective upgrades.

### 3.2. Blank Control Group Experiments

**Figure 2:**
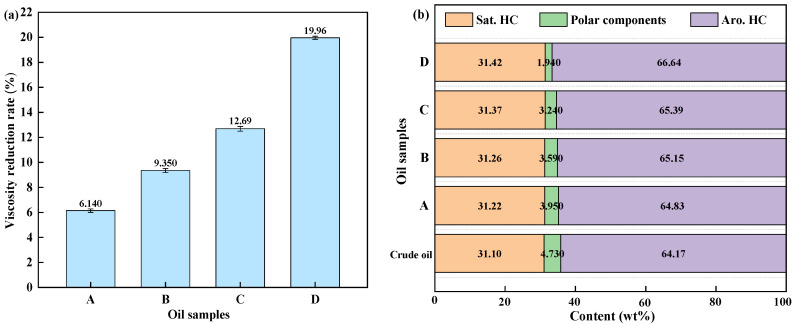
Effect of experimental conditions on viscosity reduction rate (**a**) and group composition (**b**). (A. blank; B. upgrade oil with corn stalk; C. upgrade oil with catalyst; D. upgrade oil with catalyst and corn stalk).

The viscosity reduction rates and changes in the group composition of the upgrade oil under different reaction conditions are presented in Figure 2. The aquathermolysis of FEO without a catalyst or biomass led to only a 6.140% viscosity reduction rate, with minimal changes in group composition compared to the crude oil. This finding suggests that the hydrothermal environment alone has a limited upgrading effect on this PAH-rich feedstock. During the aquathermolysis process, the primary role of the catalyst is to activate C–S bonds, C–N bonds, and unsaturated bonds in the heavy components. Corn stalk alone produced a smaller improvement than the catalyst. Notably, the combined catalyst and corn stalk system achieved the highest viscosity reduction (19.96%). In this case, the polar fraction decreased to 1.940 wt% and the aromatic–hydrocarbon fraction increased to 66.64 wt%, highlighting a synergistic effect in promoting the cleavage and stabilization of heavy fractions in FEO.

### 3.3. Analysis of Oil Samples

The viscosity of the FEO was found to be further reduced under the combined action of corn stalk and catalyst, inevitably altering its properties and composition. Hence, to elucidate the changes induced in the FEO, a comprehensive set of analyses was conducted, including molecular weight determination, elemental analysis, ^1^H NMR spectra, and detailed hydrocarbon composition characterization. These analyses were performed on the crude oil, upgrade oil obtained without corn stalk (catalyst only), and upgrade oil obtained with corn stalk (corn stalk and catalyst added simultaneously).

#### 3.3.1. ARI Analysis of Oil Samples

The aromatic ring index (ARI) characterizes the number of condensed aromatic rings in oil molecular structures [35]. The method under discussion enables a clear differentiation of distinct hydrocarbon families through the exclusive measurement of molecular weight and the refractive index. Specifically, ARI can distinguish hydrocarbon groups, including *n*-alkanes, naphthenes, benzene derivatives, and naphthalene derivatives. The ARI value of *n*-alkanes is 0, that of naphthalene derivatives is 2, and that of benzene derivatives is 0.95. ARI is estimated using Equations (5) and (6):

(5) $$ARI=f\left(M_{W},F_{RI,20}\right)=\frac{2[\frac{M_{W}}{F_{RI,20}}-(3.5149M_{W}+73.1858)]}{\left(3.5074M_{W}-91.972)-(3.5149M_{W}+73.1858\right)}$$

(6) $$F_{RI,20}=\frac{n_{20}^{2}-1}{n_{20}^{2}+2}$$

where *n*_20_ is the refractive index of the oil samples at 20 °C; *M_W_* is the molecular weight of the oil samples, g·mol^−1^; and *F_RI_* is the function of the refractive index.

Physical constants of hydrocarbon mixtures correlate with their structural units. Among the major hydrocarbon families, aromatics generally exhibit the highest density and refractive index, which increase with the number of aromatic rings. Alkanes have the lowest density and refractive index, while naphthenes fall between the two. As demonstrated in Table 5, several physicochemical properties of FEO changed after aquathermolysis. The *M*_w_ decreased to 370.4 g·mol^−1^ for the upgrade oil without corn stalk and further decreased to 353.3 g·mol^−1^ when corn stalk was added, indicating enhanced molecular lightening. The density decreased from 1.008 g·cm^−3^ to 0.9922 g·cm^−3^, suggesting a lower proportion of high-molecular-weight and high-polarity components. Despite the negligible decline in the refractive index from 1.561 to 1.557, this shift mirrors a reduction in the presence of aromatic ring systems and conjugated structures within the oil. Meanwhile, the ARI decreased from 3.049 to 2.593, indicating a reduction in aromatic ring condensation. Together, these results support a shift in product distribution toward lighter molecules with a lower degree of aromatic condensation.

#### 3.3.2. Elemental Analysis of Oil Samples

The elemental analysis results are shown in Table 6. Compared with crude oil, the upgrade oil with corn stalk exhibited a significant increase in oxygen content, whereas the oil processed without corn stalk showed a marked decrease. In addition, the contents of carbon, nitrogen, and sulfur in the upgrade oil decreased, while the contents of hydrogen increased. The upgrade oil obtained with corn stalk exhibits the highest hydrogen content and H/C ratio, demonstrating an enhanced hydrogen enrichment effect. Importantly, the H/C ratio of the physical mixture of bio-oil derived from corn stalk hydrothermal liquefaction and upgrade oil without corn stalk was 1.457, which was comparable to that of the upgrade oil without corn stalk. This indicates that the improvement cannot be explained by simple blending. Instead, catalytic co-aquathermolysis likely promotes chemical interactions between biomass-derived intermediates and FEO, thereby improving oil quality.

#### 3.3.3. ^1^H NMR Analysis of Oil Samples

**Figure 3:**
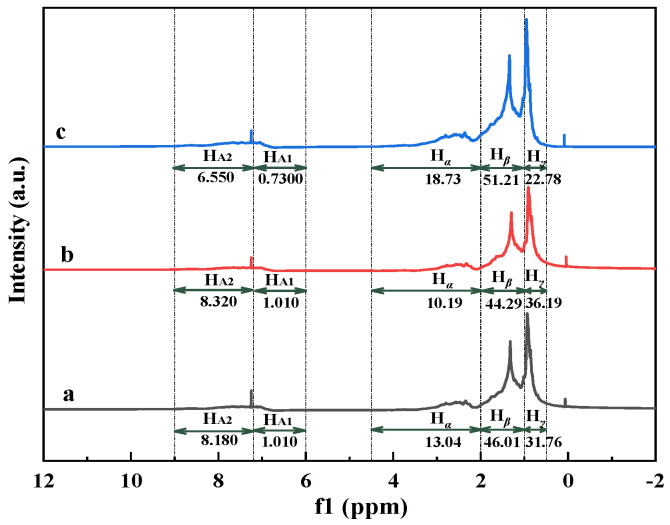
^1^H NMR spectra of oil samples: (a) crude oil; (b) upgrade oil without corn stalk; (c) upgrade oil with corn stalk.

To quantify changes in hydrogen distribution after aquathermolysis, ^1^H NMR spectra were acquired for crude and upgrade oils. The corresponding spectra are presented in Figure 3. According to the assignment of protons in the ^1^H NMR spectra (as shown in Table 4), the distribution of hydrogen attribution to different chemical shifts obtained by the area integral is displayed in Table 7.

The aromatic hydrogen (H_A_) slightly increases in the catalyst-only system, whereas it decreases markedly when corn stalk is introduced. This indicates that biomass addition suppresses aromatization/condensation and promotes the hydrogenation and stabilization of reactive fragments. Corn stalk addition also increases the aliphatic hydrogen fraction, mainly through higher H*_α_* and H*_β_* contributions, consistent with side-chain scission and the formation of more saturated fragments. For *f*_A_, a lower value indicates the less aromatic hydrocarbons. The *f*_A_ values had the following order: crude oil (0.3610) > upgrade oil without corn stalk (0.3380) > upgrade oil with corn stalk (0.2730). For H_AU_/C_A_, a higher H_AU_/C_A_ is indicative of a lower condensation degree of aromatic ring systems [36]. The order of H_AU_/C_A_ is as follows: upgrade oil with corn stalk (0.9540) > upgrade oil without corn stalk (0.6230) > crude oil (0.6110). It can be seen from the above results that the *f*_A_ value of the upgrade oil with corn stalk is the smallest and the H_AU_/C_A_ value is the largest, indicating that the aromatic ring content is reduced and the degree of aromatic condensation is weakened. This may be because in catalytic aquathermolysis, the hydrothermal liquefaction by-products of corn stalk (phenols, ketones, alcohols, etc.) provide active hydrogen on the Ni/Mo catalytic sites. This promoted hydrogenation and the ring opening of polycyclic aromatic hydrocarbons while concurrently suppressing secondary polycondensation. These trends are consistent with the biomass-assisted aquathermolysis reported previously [34]. The increase in BI in the catalyst-only system suggests side-chain isomerization and the ring opening of heterocycles during aquathermolysis. Notably, the BI of the upgrade oil with corn stalk was lower than that of the upgrade oil without corn stalk, indicating that biomass-derived hydrogen radicals preferentially promote the depolymerization and cleavage of bridge bonds in condensed ring systems [37].

#### 3.3.4. Detailed Hydrocarbon Composition Analysis of Oil Samples (GC-MS Method)

**Figure 4:**
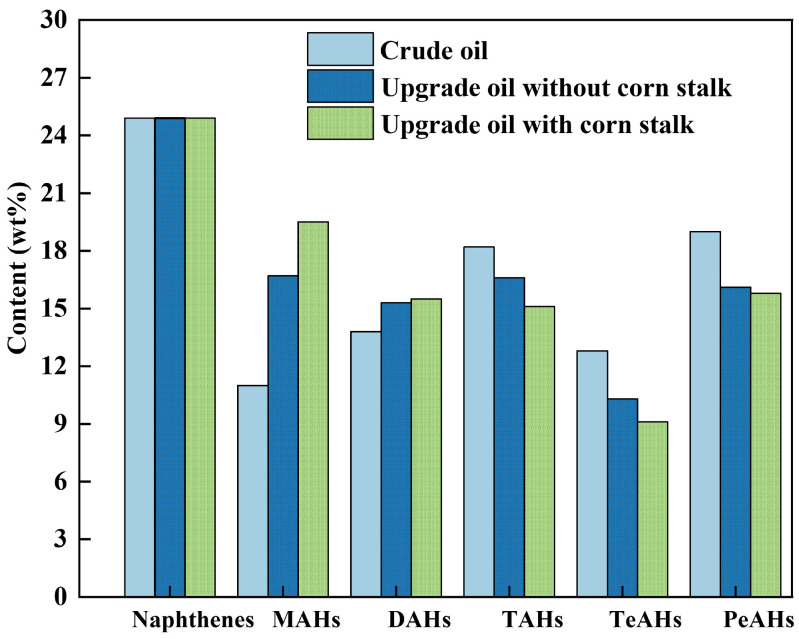
Detailed hydrocarbon composition analysis of oil samples.

The sulfur content of the crude oil is low; sulfur species were mainly thiophene sulfides and were grouped with structurally similar hydrocarbons to simplify the hydrocarbon-class analysis [38]. The detailed hydrocarbon composition is shown in Figure 4. The crude oil is dominated by tri-/tetra-aromatics and polycyclic aromatics, with a naphthene content of 24.90 wt%. The tricyclic aromatic hydrocarbons (TAHs) and tetracyclic aromatic hydrocarbons (TeAHs) are 18.20 wt% and 12.80 wt%, respectively, indicating a high proportion of heavy condensed aromatics in its structure. After upgrading without corn stalk, the naphthene content shows little change, while TAHs and TeAHs decreased slightly and low-ring aromatics increased. In contrast, when upgrading with corn stalk, the contents of TAHs and TeAHs further decreased to 15.10 wt% and 9.100 wt%, respectively, while the contents of diaromatic hydrocarbons (DAHs) and monoaromatic hydrocarbons (MAHs) further increased to 15.50 wt% and 19.50 wt%, respectively. This redistribution reflects a ring number reduction and molecular lightening rather than net aromatization of the upgrade oil.

^1^H NMR and GC–MS provide complementary but fundamentally different information. ^1^H NMR reflects the bulk hydrogen distribution of the entire upgrade oil, whereas GC-MS mainly characterizes the GC-elutable fraction. Together, these results indicate that co-aquathermolysis shifts the product distribution toward lighter fractions with a lower degree of polymerization and condensation.

### 3.4. Aqueous Phase Product Analysis

**Figure 5:**
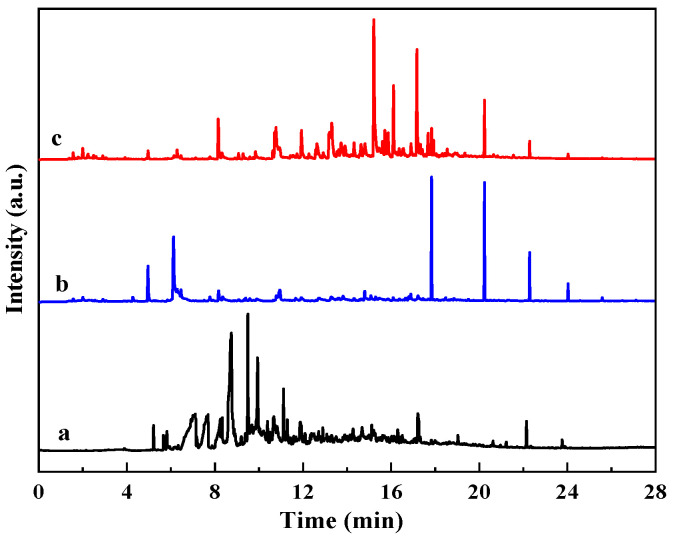
GC-MS spectra of organic matter in aqueous phase after reaction: (a) hydrothermal liquefaction of corn stalk aqueous phase; (b) modified aqueous phase without corn stalk; (c) modified aqueous phase with corn stalk.

The GC-MS spectra of organic matter in the aqueous phase after a reaction is shown in Figure 5. Compared with aquathermolysis without corn stalk, the corn-stalk-assisted system exhibited a higher peak density and larger total peak area, indicating an increased concentration of dissolved organics. Peak clusters assigned to furans, cyclic ketones, and phenols appear mainly at 6–12 min and 15–22 min, whereas signals in the heavy fraction (>22 min) were attenuated. The aqueous phase obtained from corn stalk hydrothermal liquefaction shows a similar distribution, suggesting that biomass liquefaction products contribute substantially to the aqueous organics during co-aquathermolysis. These oxygenated species can participate in hydrogen transfer reactions, increasing reactive hydrogen availability and suppressing radical recombination and secondary polycondensation.

### 3.5. Characterization of Catalysts

To verify the catalyst’s stability and its interaction with biomass-derived intermediates during co-aquathermolysis, post-reaction catalyst characterization was performed using FT-IR, SEM, and EDX.

#### 3.5.1. FT-IR Analysis

**Figure 6:**
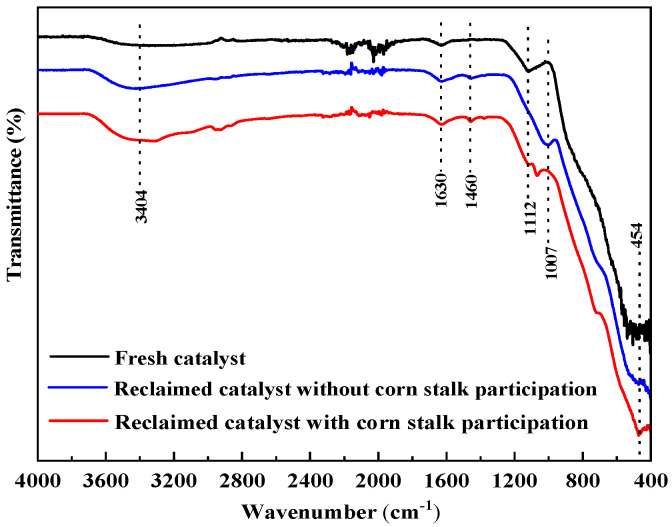
FT-IR spectra of catalyst.

Fourier transform infrared (FT-IR) spectra were used to investigate the chemical structure of the catalysts, and the results are shown in Figure 6. The peaks at 3404 and 1630 cm^−1^ may originate from various vibrations of H_2_O molecules adsorbed on the catalyst surface [39]. In the fingerprint region, the absorption peaks at 1112 cm^−1^ are attributed to the stretching vibrations of the Al–O bond [40]. The peaks observed at 1007 and 454 cm^−1^ are attributed to Mo=O stretching and Ni-O lattice vibrations, respectively [41]. After the reaction, the positions of the metal–oxygen vibration bands remain nearly unchanged. However, a novel absorption peak emerges at 1460 cm^−1^, attributable to surface carbonate species formed at metal oxide sites. This result indicates the enhanced adsorption and stability of CO_2_ and oxygen-containing species during aquathermolysis, particularly when corn stalk is added.

#### 3.5.2. SEM and EDX Analysis

**Figure 7:**
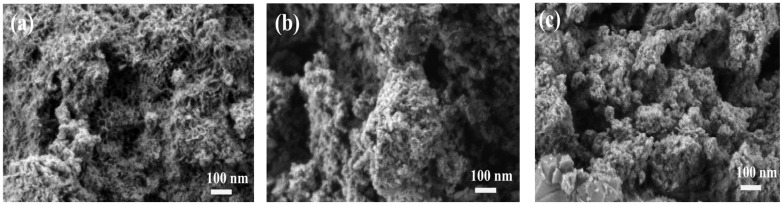
SEM images of the catalyst: (**a**) fresh catalyst; (**b**) reclaimed catalyst without corn stalk participation; (**c**) reclaimed catalyst with corn stalk participation.

A SEM image of the catalyst is shown in Figure 7. The fresh catalyst (Figure 7a) exhibits a loose and highly porous morphology with abundant interparticle voids and a rough surface texture. After a reaction without corn stalk participation (Figure 7b), the reclaimed catalyst exhibits particle agglomeration and structural densification. The original loose, porous structure was partially replaced by larger, block-like aggregates and the pore contours become indistinct. In contrast, the catalyst recovered with corn stalk participation (Figure 7c) shows an even more compact and irregular morphology. The aggregates appear more tightly packed, and the porous features are further obscured, suggesting more severe deposition-induced pore blocking.

**Figure 8:**
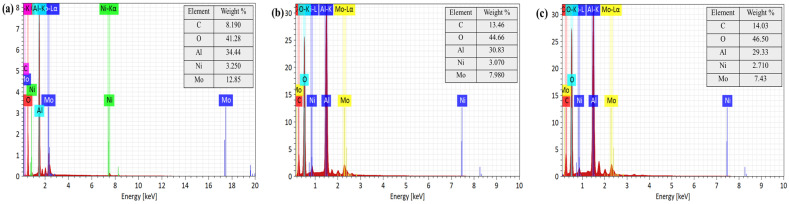
EDX spectra with elemental distribution of catalyst: (**a**) fresh catalyst; (**b**) reclaimed catalyst without corn stalk participation; (**c**) reclaimed catalyst with corn stalk participation.

The EDX spectra with the elemental distribution of the catalyst are shown in Figure 8. All samples exhibited characteristic signals of aluminum and oxygen from the alumina support as well as nickel and molybdenum from the active components. Compared with the fresh catalyst, the reclaimed catalysts showed increased surface carbon accompanied by higher oxygen signals and decreased apparent nickel and molybdenum contents. Together with the SEM observations, these changes indicate the formation of a deposit layer and intensified agglomeration after the reaction, which progressively covered the catalyst surface and partially masked nickel and molybdenum signals. This effect was more pronounced when corn stalk was present.

### 3.6. Mechanism Analysis

Under hydrothermal conditions, PAH-rich FEO undergoes thermal and catalytic activation. Operating under a near-saturated vapor–liquid regime at 280 °C maintains a high-density aqueous phase while allowing for limited vapor formation. Under such conditions, hot compressed water exhibits an enhanced ion product and solvation capacity, which facilitates bond cleavage, radical generation, and hydrogen transfer reactions. The cleavage of heteroatom-containing bonds (e.g., C–S, C–N) and side chains generates radical intermediates (alkyl radicals R• and aryl radicals Ar•). Over the Ni/Mo hydrofining catalyst, metal oxide centers promote redox-assisted bond activation and hydrogenation, while acidic sites on the alumina support facilitate cracking and isomerization, thereby enabling structural rearrangement. In the absence of an external hydrogen donor, the supply of reactive hydrogen derived from water is limited. This allows for radical recombination and polycondensation to compete with stabilization, resulting in incomplete molecular weight reduction.

With the addition of corn stalk, hydrothermal liquefaction produces oxygenates (e.g., phenols, alcohols, ketones, and organic acids) and light gases (e.g., CO), which are readily activated on the Ni/Mo sites. This process enhances the generation of reactive hydrogen species (H•) through pathways such as water dissociation, the water–gas shift reaction, and dehydrogenation/reforming. The resulting H• rapidly quenches radical fragments (H• + R• → R-H), suppressing secondary polycondensation and stabilizing cracked intermediates. Consequently, hydrogen transfer reactions shift condensed aromatics toward lower-ring aromatics and partially hydrogenated products.

The proposed mechanism is further supported by post-reaction catalyst characterization. As shown in the FT-IR spectra (Figure 6), the preservation of Ni–O and Mo=O vibration bands indicates that the metal oxide active phases remain structurally stable during co-aquathermolysis, while the emergence of carbonate-related bands suggests the adsorption and transformation of oxygen-containing intermediates derived from biomass and water. In addition, SEM and EDX analyses (Figure 7 and Figure 8) reveal increased surface carbon deposition and partial coverage of Ni/Mo sites after a reaction, particularly in the presence of corn stalk, confirming strong interactions between biomass-derived species and the catalyst surface. These observations are consistent with the proposed hydrogen transfer and radical stabilization pathways.

## 4. Conclusions

In this study, a series of co-aquathermolysis experiments were conducted to systematically investigate the effects of reaction conditions on the upgrade efficiency of FEO. In addition, the influence of corn stalk on the properties of co-aquathermolysis products was analyzed in depth. The main conclusions are listed below.

(1) The optimal upgrade efficiency was achieved under the following conditions: 280 °C, 18 h reaction time, and 8 wt% dosage for both the catalyst and corn stalk. Under these conditions, the viscosity reduction rate of the FEO reached 19.96%, which was significantly superior to that obtained without corn stalk.

(2) After upgrading, the average molecular weight of the FEO decreased from 430.0 to 353.3 g·mol^−1^, while the density and refractive index decreased slightly and the aromatic ring index dropped from 3.049 to 2.593. Elemental analysis showed that the H/C ratio increased from 1.407 to 1.568 and the sulfur content decreased from 0.1130% to 0.09210%, indicating that the FEO underwent a certain degree of upgrades, hydrogenation, and desulfurization. ^1^H NMR analysis confirmed the cleavage of long-chain hydrocarbons and a reduction in the aromatic condensation degree and aromatic carbon fraction. Detailed hydrocarbon composition analysis further confirmed the conversion of tricyclic and tetracyclic aromatic hydrocarbons to monocyclic and bicyclic aromatic hydrocarbons, which verified the depolymerization and lightening trends of macromolecular structures during the upgrade process.

(3) During the coupled upgrade process, hydrothermal liquefaction products of corn stalk (phenols, ketones, alcohols, etc.) generated active hydrogen via catalysis by the Ni/Mo metal sites, thereby capturing the free radical fragments produced from FEO cracking, effectively inhibiting secondary polycondensation reactions and realizing the hydrocracking of condensed aromatics and quality improvement of FEO.

## Figures and Tables

**Table 1:** Fundamental physical characteristics of furfural extraction oil.

Property	Value
Density/(20 °C, g·cm^−3^)	1.008
Refractive index/(20 °C)	1.561
Kinematic viscosity (100 °C)/(mm^2^·s^−1^)	57.22
Dynamic viscosity (40 °C)/(mPa·s)	978.3
Molecular weights (*M*_W_)/(g·mol^−1^)	430.0
Aromatic carbon ratio/(%)	32.80
Naphthenic carbon ratio/(%)	40.10
Paraffinic carbon ratio/(%)	27.10
Saturated hydrocarbons/(wt%)	31.10
Aromatic hydrocarbons/(wt%)	64.17
Resins/(wt%)	4.710
Asphaltenes/(wt%)	0.02000

**Table 2:** Fundamental physical characteristics of corn stalk.

Property	Value
Cellulose content/(wt%)	22.30
Hemicellulose content/(wt%)	35.10
Lignin content/(wt%)	14.80
C/(wt%)	39.16
H/(wt%)	5.979
S/(wt%)	0.2420
N/(wt%)	0.9000
O/(wt%)	53.72

**Table 3:** Fundamental properties of catalyst.

Property	Value
S_BET_ ^a^/(m^2^·g^−1^)	247.3
V_P_ ^b^/(cm^3^·g^−1^)	0.2118
D_P_ ^c^/(nm)	8.244
Content of strong acid sites/(mmol·g^−1^)	0.1500
Content of weak acid sites/(mmol·g^−1^)	1.840
NiO loading amount/(wt%)	7.761
MoO_3_ loading amount/(wt%)	28.55

^a^ BET specific surface area; ^b^ BJH pore volume; ^c^ BJH average pore diameter.

**Table 4:** The assignment of protons in ^1^H NMR spectra.

Parameter	Type of Protons	Chemical Shifts (ppm)
H_A_	aromatic hydrogen	6.0–9.0
H_A1_	aromatic hydrogens in monoaromatic structures	6.0–7.2
H_A2_	aromatic hydrogens in polyaromatic structures	7.2–9.0
H*_α_*	aliphatic hydrogen on C*_α_* to aromatic rings	2.0–4.5
H*_β_*	aliphatic hydrogen on C*_β_* and CH_2_; CH beyond C*_β_* to aromatic rings	1.0–2.0
H*_γ_*	aliphatic hydrogen on C*_γ_* and CH_3_ beyond C*_γ_* to aromatic rings	0.5–1.0

**Table 5:** ARI analysis of oil samples.

Samples	*M*_W_/(g·mol^−1^)	*ρ*_20/_(g·cm^−3^)	*n*_20_	ARI
Crude oil	430.0	1.008	1.561	3.049
Upgrade oil without corn stalk	370.4	0.9998	1.559	2.714
Upgrade oil with corn stalk	353.3	0.9922	1.557	2.593

**Table 6:** Elemental composition of oil samples.

Samples	C/(wt%)	H/(wt%)	S/(wt%)	N/(wt%)	O */(wt%)	H/C
Crude oil	87.78	10.29	0.1130	0.4910	1.326	1.407
Upgrade oil without corn stalk	87.54	10.65	0.09820	0.4701	1.242	1.460
Upgrade oil with corn stalk	86.66	11.32	0.09210	0.4701	1.458	1.568
Hydrothermal liquefaction bio-oil from corn stalk	68.52	6.860	0.08530	1.190	23.34	1.201
Bio-oil and upgrade oil without corn stalk	87.24	10.59	0.09800	0.4816	1.590	1.457

*: oxygen calculated by difference.

**Table 7:** The parameters determined from ^1^H NMR spectra.

Samples	Crude Oil	Upgrade Oil Without Corn Stalk	Upgrade Oil with Corn Stalk
H_A_ (%)	9.190	9.330	7.280
H_A1_ (%)	1.010	1.010	0.7300
H_A2_ (%)	8.180	8.320	6.550
H_α_ (%)	13.04	10.19	18.73
H_β_ (%)	46.01	44.29	51.21
H_γ_ (%)	31.76	36.19	22.78
C_T_/H_T_	0.7110	0.6850	0.6380
*f*_A_	0.3610	0.3380	0.2730
H_AU_/C_A_	0.6110	0.6230	0.9540
BI	0.3590	0.4430	0.2170

## Data Availability

The original contributions presented in the study are included in the article. Further inquiries can be directed to the corresponding author.

## Supplementary Materials

The following supporting information can be downloaded at: https://www.mdpi.com/article/10.3390/ma19020428/s1, Figure S1: Temperature–pressure–time profiles of a representative run at 280 °C under autogenous pressure.
