# Supplementary material for: Study on Synergistic Viscosity Reduction Mechanism and Product Characteristics of Co-Aquathermolysis of Corn Stalk and Furfural Extraction Oil
Source: Materials (Basel). 2026 Jan 22;19(2):428. doi: 10.3390/ma19020428 (PMC12843359; doi:10.3390/ma19020428)
Supplement: Supplementary file 1 [file materials-19-00428-s001.zip › materials-4036254-supplementary-done.pdf]

materials

Supplemental material

Study on the Synergistic Viscosity Reduction Mechanism and Product Characteristics of Co-Aquathermolysis of Corn Stalk and Furfural Extraction Oil

Qingmei Tian [1,2], Zinan Liu [3], Wenqiang Liu [2], Yansheng Liu [1,2], Xingying Lan [4] and Xiaoling Xu [2,*]

1 College of Chemical Engineering and Environment, China University of Petroleum (Beijing), Beijing 102249, China; tqm@cupk.edu.cn (Q.T.); wsuper@cup.edu.cn (Y.L.)
2 State Key Laboratory of Heavy Oil Processing, China University of Petroleum (Beijing) at Karamay, Karamay 834000, China; 15811385563@163.com
3 Refining and Chemical Research Institute, PetroChina Karamay Petrochemical Company Limited, Karamay 834000, China; 18209902736@163.com
4 State Key Laboratory of Heavy Oil Processing, China University of Petroleum (Beijing), Beijing 102249, China; lanxy@cup.edu.cn
* Correspondence: xuxiaoling@cupk.edu.cn.

Figure S1. Temperature–pressure–time profiles of a representative co-aquathermolysis run conducted in a sealed batch reactor under autogenous pressure (oil-to-water mass ratio 2:1, 280 °C, 18 h, 8 wt% catalyst, 8 wt% corn stalk). The pressure increased with temperature during heating, reached 6.41 MPa at 280 °C, and remained stable during the isothermal stage, indicating near-saturated vapor–liquid operation.

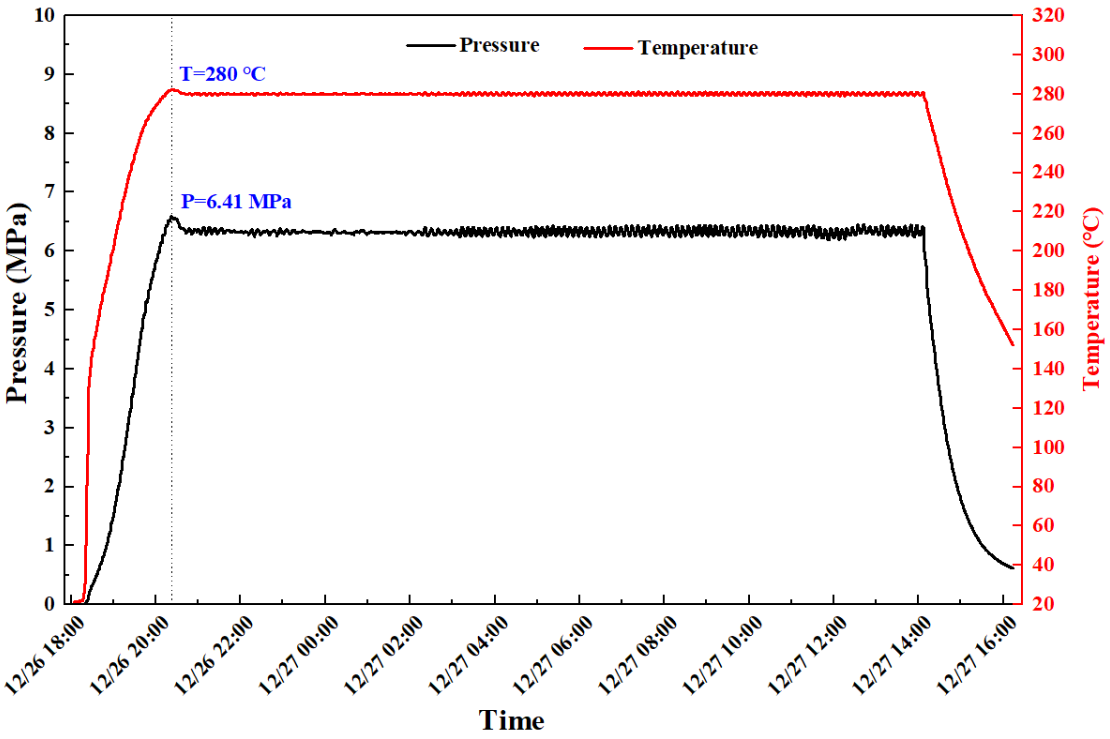

Figure S1. Temperature–pressure–time profiles of a representative run at 280 °C under autogenous pressure.

 https://doi.org/10.3390/ma19020428
